# Activation of p38/JNK Pathway Is Responsible for Embelin Induced Apoptosis in Lung Cancer Cells: Transitional Role of Reactive Oxygen Species

**DOI:** 10.1371/journal.pone.0087050

**Published:** 2014-01-22

**Authors:** Deepa R. Avisetti, K. Suresh Babu, Shasi V. Kalivendi

**Affiliations:** 1 Centre for Academy of Scientific & Innovative Research, CSIR-Indian Institute of Chemical Technology (CSIR-IICT), Hyderabad, Andhra Pradesh, India; 2 Centre for Chemical Biology, CSIR-Indian Institute of Chemical Technology (CSIR-IICT), Hyderabad, Andhra Pradesh, India; 3 Natural Products Chemistry, CSIR-Indian Institute of Chemical Technology (CSIR-IICT), Hyderabad, Andhra Pradesh, India; University of Kansas School of Medicine, United States of America

## Abstract

The natural product embelin has been demonstrated to possess a wide range of therapeutic properties, however, the mechanisms by which it exerts anticancer effects are not yet clear. By monitoring the molecular changes associated during early apoptotic phase, we have identified the crucial role of oxidative stress induced MAP kinase signalling as a predominant mechanism for its anticancer effects. Treatment of A549 lung cancer cells with embelin resulted in the enhancement of phospho-p38 and phospho-JNK levels as early as 4h. Pretreatment of cells with specific inhibitors of p38 (PD169316) and JNK (SP600125) abrogated embelin-induced caspase-3 activation. Studies employing embelin in the presence or absence of specific MAP kinase inhibitors indicated that the observed changes in phosphorylation levels of p38, JNK and ERK 1/2 are solely due to embelin and not because of cross-talk between MAP kinases. Reactive oxygen species (ROS) play a crucial role in embelin induced alterations in MAP kinase phosphorylation and apoptosis as pretreatment of cells with FeTMPyP mitigated this effect. The observed changes are not due to the inhibitory effect of embelin on XIAP as cells treated with SMAC-N7-Ant peptide, a specific inhibitor of XIAP’s BIR3 domain did not mimic embelin induced apoptotic effects. The findings of the present study clearly indicate the crucial role of p38 and JNK pathways in embelin induced apoptosis and provide us with new clues for improving its therapeutic efficacy.

## Introduction

Embelin, an active component of fruits of *Embelia ribes,* has been demonstrated to possess a broad-spectrum of therapeutic properties such as anticancer, anti-inflammation, anti-diabetes, anti-obesity, analgesic, anti-fertility and anti-helminthic [1]–[5]. The initial discovery of embelin as an inhibitor of XIAP by virtue of its interaction with the BIR3 domain and its observed selectivity towards cancer cells as compared to the normal cells inspired us to consider it as a lead compound for further studies against cancer [6]. As many of the cancers express elevated levels of XIAP and become refractory to apoptosis, treatment with embelin or in combination with other known anticancer drugs was found to sensitize them towards apoptosis [6], [7]. Mechanism based studies indicate that embelin inactivates NF-kB by inhibiting nuclear transportation of p65 and also shown to inhibit STAT3 phosphorylation by inducing the expression of PTEN [8], [9].

Specific efforts to identify the precise molecular target of embelin resulted in the identification of embelin as an inhibitor against XIAP’s BIR3 domain [6]. In addition, embelin was also demonstrated to be an inhibitor of 5-lipoxigenase and microsomal prostaglandin E2 synthase-1 (mPGES)-1; plasminogen activator inhibitor-1 (PAI-1) and P300/CBP associated factor (PCAF) [10]–[12]. Moreover, embelin has been shown to interfere with the oxidative phosphorylation of mitochondria and can undergo both redox and non-redox mediated mechanisms [13], [14].

Though the affinity of embelin against some of the molecular targets and cell signalling mechanisms have been identified, the primary intracellular target responsible for its anti-cancer property is not yet clear as many of the earlier studies have been carried out at later time points where the signal transduction cascade becomes complex due to the cross-talk between multiple cell signalling mechanisms [8], [15], [16], [17]. Hence, in the present study, we sought to identify the alterations in signalling pathways responsible for the anticancer property of embelin during the early apoptotic phase. The present study identified for the first time the pivotal role of MAP kinase pathway, especially p38 and JNK, in embelin induced apoptosis.

## Materials and Methods

### Materials

Embelin was purified from the fruits of *Embelia ribes* as described previously [18], [19]. Minimal essential medium (MEM), Dulbecco’s modified Eagle’s medium (DMEM), Dulbecco’s phosphate buffered saline (DPBS), penicillin, streptomycin, sulphorhodamine B (SRB), Ac-DEVD-7-AFC, Ac-LEHD-7-AFC, PD169316, SP600125, N-acetyl-L-cysteine (NAC), radioimmune precipitation assay buffer (RIPA) and protease inhibitor cocktail were purchased from Sigma-Aldrich, Germany. U0126 and FeTMPyP were purchased from Calbiochem. SMAC-N7-Ant peptide (AVPIAQK-P-RQIKIWFQNRRMKWKK) was synthesized by GenPro Biotech, Noida, India. Annexin-V assay kit was purchased from Clontech Inc, USA. All the chemicals for buffer preparations and fine chemicals were purchased from Sigma-Aldrich, Germany.

### Cell Culture and Experimental Conditions

All the cell lines were obtained from ATCC, USA. A549, DU145, MCF-7 and WPMY-1 cells were grown in MEM (supplemented with 10% FBS, 100 units/ml penicillin and 100 units/ml streptomycin) while H9c2 and MRC-5 cells were grown in DMEM (supplemented with 10% FBS, 100 units/ml penicillin and 100 units/ml streptomycin). Cells were maintained in humidified atmosphere with 5% CO2 at 37°C. Twelve hours before treatments, the cell culture media was replaced with respective media containing 2% FBS, unless otherwise indicated. In intervention studies, cells were pretreated with the respective MAP kinase inhibitors or antioxidants for 1h before the addition of embelin (15 µM). For experiments involving SMAC-N7-Ant peptide, cells were treated with 100 µM peptide for a period of 8h.

### Cytotoxicity Assay

The effect of embelin on cell viability was determined by sulphorhodamine B (SRB) assay as described previously [20]. SRB is an aminoxanthene dye that binds to basic amino acid residues of cells (fixed to tissue culture plates by trichloroacetic acid) under mild acidic conditions [20]. Briefly, cells (in 24 well plates, ∼ 80% confluence) were treated with different concentrations of embelin for 48h in media supplemented with 10% fetal bovine serum. Following the termination of incubation, cells were fixed by the addition of 30% trichloroacetic acid to the medium at 4°C for 1h. Later, cells were washed with deionised water and air dried. SRB (0.04%, w/v) was added to the cells and incubated further for 30 min at room temperature. Finally, cells were washed with 1% acetic acid (three times) and air dried. SRB bound to the cells was solubilised in 10 mM Tris-base and absorbance was measured at 565 nm using EnSpire multimode plate reader (Perkin Elmer).

### Caspase 3 and 9 Assay

Following the termination of incubation, cellular caspase-3 and -9- activities were measured using AFC conjugated tetrapeptide substrates as described previously [21]. Briefly, cells were washed with ice-cold DPBS and lysed in ice-cold lysis buffer (50 mM HEPES pH 7.4, 5 mM CHAPS and 5 mM DTT) [22]. Lysates were pelleted down at 12,000 g for 10 min at 4°C and supernatants were collected. To the lysates equal volumes of assay buffer (40 mM HEPES pH7.4, 0.2% CHAPS, 10 mM DTT, 4 mM EDTA) containing either caspase-3 substrate (Ac-DEVD-7- AFC, 40 µM) or caspase-9 substrate (Ac-LEHD-7-AFC, 40 µM) was added and incubated at 37°C. Increase in the fluorescence readings due to the release of AFC was monitored for every 5 min interval at λex 400 nm and λem 505 nm for 1h using EnSpire multimode plate reader (Perkin Elmer). Protein estimation was performed by Bradford’s method and the fluorescence units were normalized to the total protein in the incubation mixture.

### Annexin-V/FITC Analysis for Apoptosis

A549 cells grown on coverslips in 6-well plates were treated with embelin for 4h. After treatment, coverslips were washed twice with PBS followed by 1X binding buffer. Cells were then stained with annexin-V/FITC antibody by incubating in the dark for 30 min and washed with 1X binding buffer to remove any unbound antibody as per the Manufacturer’s protocol (Clontech Inc, USA). Formaldehyde (2%) was added to fix the cells at the end of incubation. Fluorescence was monitored using an Olympus-IX71inverted microscope equipped with FITC and rhodamine filter settings.

### Gene Expression Profiling using Microarray

A549 cells were treated with embelin for 4h. Following treatments, RNA was isolated using Qiagen’s kit as per the manufacturer’s instructions. The concentration and purity of the RNA extracted were evaluated using the Nanodrop Spectrophotometer (Thermo Scientific). The integrity of the extracted RNA was analyzed on the Bioanalyzer (Agilent). RNA was considered to be of good quality based on the 260/280 values, rRNA 28S/18S ratios and RNA integrity number (RIN). The samples were labeled using Agilent Quick Amp Kit. 500 ng of total RNA was reverse transcribed using oligo-dT primer tagged to T7 promoter sequence. cDNA thus obtained was converted to double stranded cDNA in the same reaction. Further the cDNA was converted to cRNA in the *in vitro* transcription step using T7 RNA polymerase enzyme and Cy3 dye was added into the reaction mix. cRNA obtained was cleaned up using RNeasy columns (Qiagen Inc) and the concentration and amount of dye incorporated was determined using Nanodrop. The specific activity for all the samples greater than 8 pmol dye/µg cRNA were considered ideal for hybridization. Labeled cRNA (600 ng) was hybridized on the array (Custom Whole Genome Human 8×60k designed by Genotypic Technology Private Limited AMADID: 027114) using the Gene Expression Hybridization kit in Sure hybridization Chambers (Agilent) at 65°C for 16h. Hybridized slides were washed using Gene Expression wash buffers. The hybridized, washed microarray slides were then scanned on a microarray scanner (G2505C, Agilent Technologies). Data extraction from images was done using Feature Extraction software and images were quantified (Version 10.7 of Agilent). Feature extracted raw data was analyzed using GeneSpring GX Version 11.5 software from Agilent. Normalization of the data was done in GeneSpring GX using the 75^th^ percentile shift. Significant genes up and down regulated showing two-fold and above within the samples with respect to control sample were identified. Statistical *t*-test p-value was calculated based on Student’s *t*-test Algorithm. Genes were classified based on functional category and pathways using GeneSpring GX and Genotypic Biointerpreter-Biological Analysis Software. The microarray data has been submitted to GEO database with accession number GSE50545.

### Intracellular ROS Measurement

Reactive oxygen species generation in cells was determined by carboxy-H2-DCFDA (Molecular Probes) as described previously [23]. Following the termination of treatments, media was aspirated and cells in 12-well plates were washed twice with DPBS. Serum free media containing 10 µM carboxy-H2-DCFDA was added to cells and incubated further at 37°C for 20 min. Finally, cells were washed twice with DPBS before adding culture medium. Intracellular fluorescence was monitored using an Olympus-IX71inverted microscope equipped with FITC filter setting.

### Western Blot Analysis

Following treatments, cells were washed with DPBS, gently scraped and collected by brief centrifugation (300 g for 3 min) and resuspended in 100 µl RIPA containing protease inhibitor cocktail and sodium ortho-vanadate, 10 mM (Sigma). The resulting cell suspension was passed through a 26 gauge needle 10 times to ensure complete lysis. The lysate was centrifuged at 12000 g for 15 min at 4°C and the clear supernatants were collected in separate tubes. Since all the antibodies employed are monoclonal, instead of stripping and reprobing the immunoblots for total and phospho-specific proteins, we have performed immunoblotting separately in order to avoid any background signals. Following protein estimation by Bradford’s method, proteins (25 µg) were resolved on 10% SDS-PAGE and blotted on to a nitrocellulose membrane. Blots were probed with monoclonal antibodies raised against total and phospho specific antibodies for ERK 1/2, p38, JNK (Cell Signaling Technology); and tubulin (Sigma-Aldrich). Anti-rabbit Ig-G conjugated to HRP (GE Life Sciences) and anti-mouse IgG conjugated to alkaline phosphatase (Sigma-Aldrich) were employed as the secondary antibodies for the MAP kinase and tubulin antibodies respectively. Bands were developed using ECL Prime Western blotting reagent (GE, Life Sciences) or BCIP/NBT reagent for tubulin (Sigma-Aldrich) and the band intensities were calculated using GeneTools software (Syngene gel documentation system).

### Statistical Analysis

All experiments were performed in triplicates and the results were expressed as mean±S.D. Statistical significance was determined by Student’s *t* test using SIGMAPLOT software.

## Results

### Embelin Exhibits Enhanced Cytotoxicity in Cancer Cells as Compared to Normal Cells

**Figure 1:**
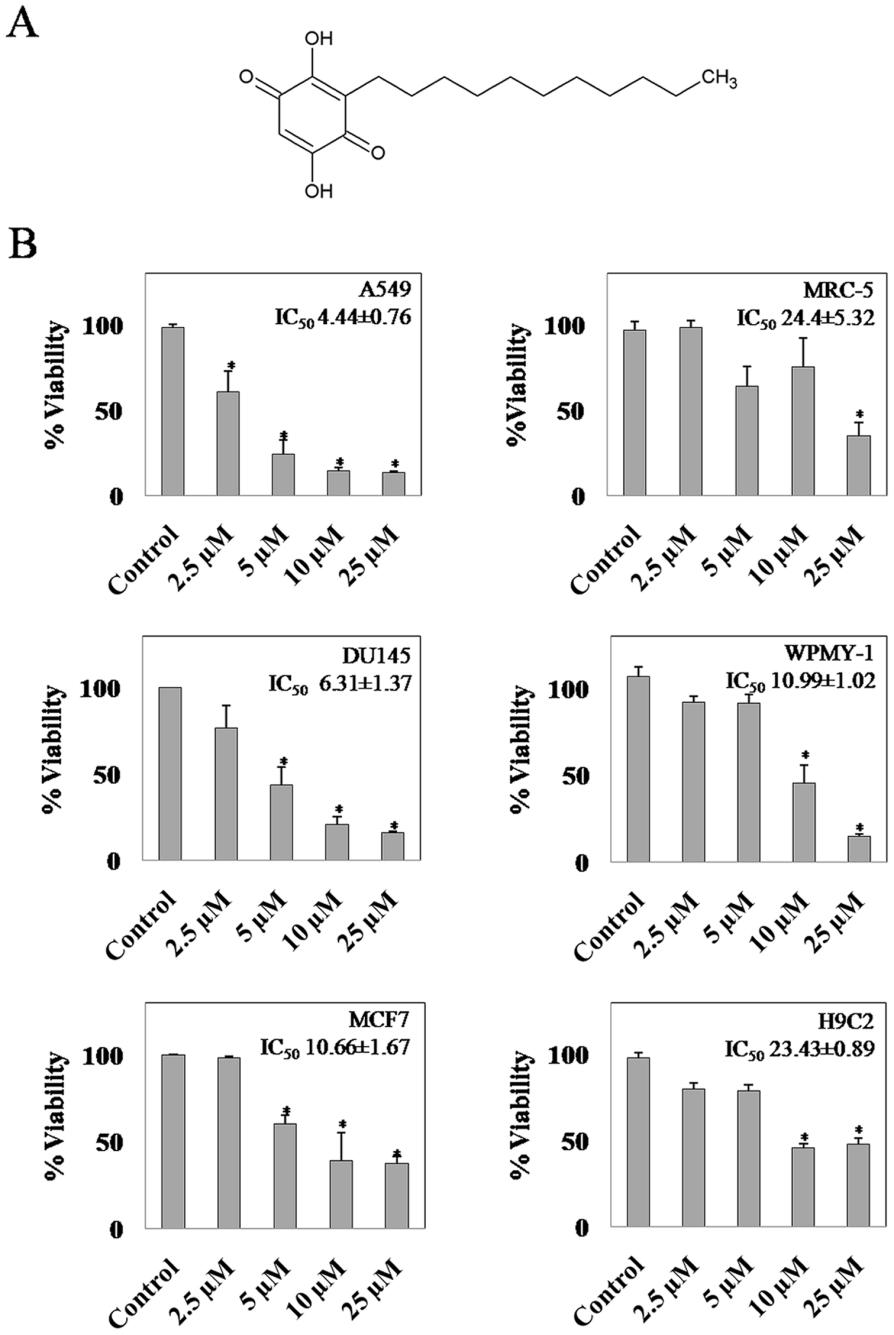
Cytotoxicity of embelin in cancer and normal cell lines. (**A**) Structure of Embelin (**B**) Cells were treated with embelin for 48h and following the termination of incubation, cell viability was measured by sulphorhodamine B assay and IC_50_ values were calculated as mentioned in the “Materials and Methods” section. Data shown are mean ± SD of three separate experiments. * indicates p<0.01as compared with controls.

**Figure 2:**
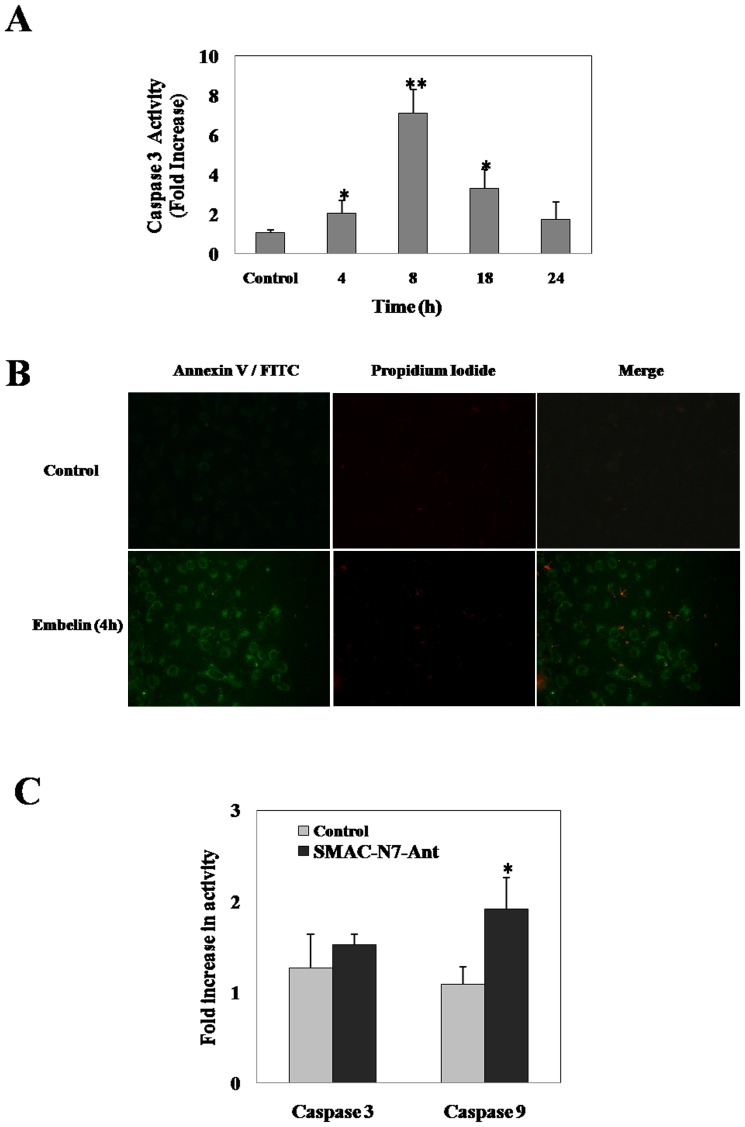
Effect of embelin and SMAC-N7-Ant peptide on cellular apoptosis. (**A**) A549 cells were treated with 15 µM embelin for different time intervals. Following the termination of treatments, caspase-3 activity was measured as indicated in the “Materials and Methods” section. (**B**) A549 cells were treated with 15 µM embelin for 4h and stained with Annexin-V/FITC and propidium iodide as described in the “Materials and Methods” section. Fluorescence images were captured using an Olympus–IX71 inverted fluorescence microscope equipped with FITC and rhodamine filter settings. Representative images from three different fields of view are shown. (**C**) Cells were treated with an XIAP inhibitor, SMAC-N7-Ant peptide (100 µM) for 8h. Later, caspase-3 and -9- activities were measured using the tetra-peptide substrates as described under “Materials and Methods” section. For both (**A**) and (**C**) data presented are the mean ± SD of three separate experiments. **indicates p<0.01 and * indicates p<0.05 as compared with controls.

The anti-proliferative activities of embelin were compared by SRB assay in selected cancer and normal cell lines (Fig. 1). Cells were subjected to increasing concentrations of embelin (2.5, 5, 10 and 25 µM) for 48h. Among the cancerous cells, embelin was found to be more toxic to A549 cells with an IC_50_ value of 4.4 µM followed by DU145 and MCF7 with 6.31 and 10.66 µM respectively. However, the observed IC_50_ values were comparatively less than the normal cell lines viz., MRC5, WPMY-1 and H9c2 which demonstrated an IC_50_ value of 24.4, 10.9 and 23.4 µM respectively. The difference between the observed IC_50_ values of lung cancer and normal cells (4.44±0.76 and 24.44±5.32 µM) appeared to be more significant. As A549 cells exhibited enhanced sensitivity towards embelin, all further studies have been carried out using this cell line for understanding the mode of action of embelin to gain novel insights to selectively target lung cancer cells as compared to their normal cell counterpart. Hence, in order to identify the early apoptotic phase, we have analyzed the time dependent effect of embelin on cellular caspase-3 activity in A549 cells (Fig. 2A). Embelin (15 µM) induced nearly 2-fold increase in the caspase-3 activity as early as 4h time period which further increased upto 6-fold by 8h (Fig. 2A). Annexin-V/FITC staining of cells treated with embelin (4h) clearly indicates the early apoptotic stage of cells as they were stained only with annexin-V but not with propidium iodide (Fig. 2B). However, at later time points i.e., 18 and 24h, the caspase-3 activities decreased nearly to 4 and 2-fold respectively indicating that apoptotic cells may have completely died by the end of 18 or 24h or there could also be a possibility of multiple cell death mechanisms due to the cross-talk between various signalling mechanisms.

As embelin is known to inhibit XIAP by binding to the BIR3 domain similar to that of SMAC, we next examined whether the observed affects of embelin on cellular apoptosis could be demonstrated by a cell permeable SMAC-N7-Ant peptide (comprised of mature SMAC’s amino terminal 7 amino acid peptide - AVPIAQK bound to Ant peptide -RQIKIWFQNRRMKWKK, for cell permeability by a proline linker) which is known to specifically interact with BIR3 domain of XIAP [24], [25]. Results demonstrate that treatment of A549 cells with SMAC-N7-Ant peptide (100 µM for 8h) increased cellular caspase-9 activity to nearly two-fold with respect to untreated control, however, no significant increase in caspase-3 activity was observed (Fig. 2C). Though the observed effects of SMAC-N7-Ant peptide are in accordance with earlier report [24], lack of caspase-3 activation with SMAC-N7-Ant peptide, but not with embelin, prompted us to investigate the responsible pathways mediating embelin induced apoptosis for gaining novel insights into its mechanism of action.

### Alteration in Gene Expression Profile by Embelin

In order to identify the pathways responsible in embelin induced apoptosis, we have analyzed the altered gene expression profile in A549 cells treated with embelin (15 µM for 4h). A total of 215 upregulated and 80 downregulated genes were identified which showed at least two-fold difference over controls with a significance of p<0.05.

**Figure 3:**
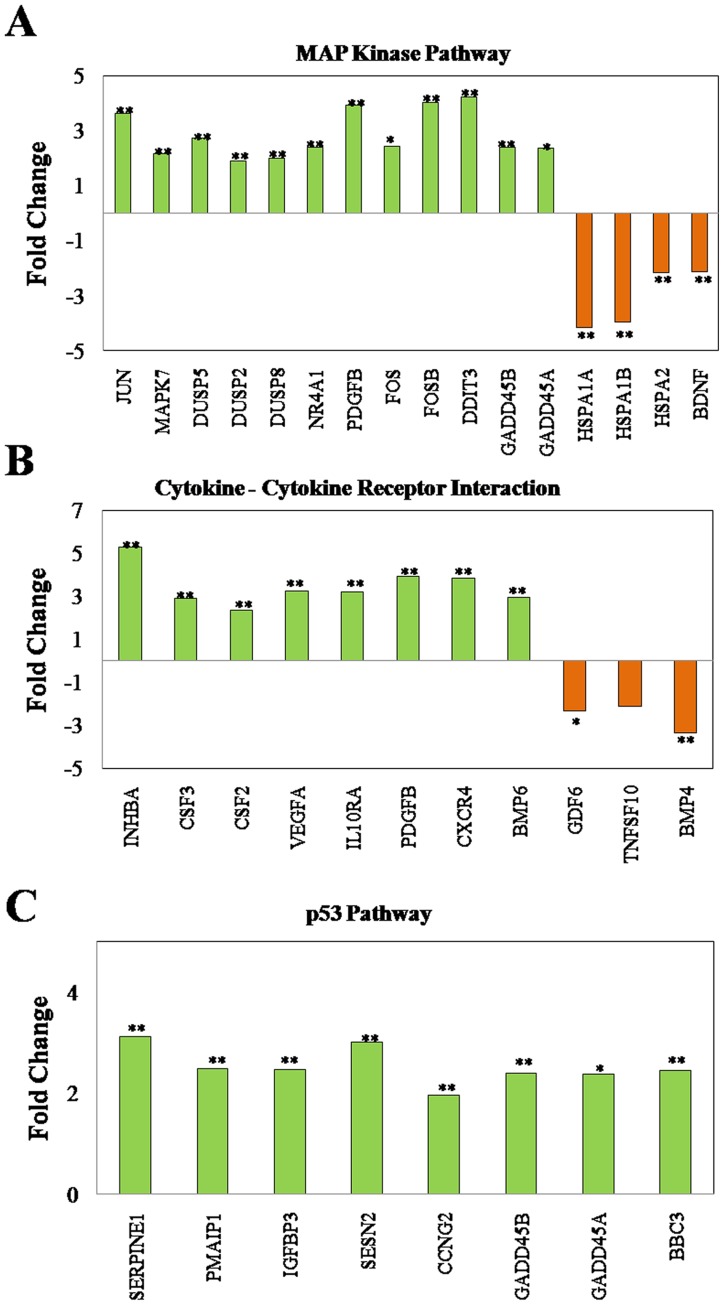
Alterations in pathway based gene expression profile induced by embelin. A549 cells were treated with embelin (15 µM) for 4h. Microarray analysis was performed as described in “Materials and Methods” section. Genes that showed differential regulation by at least 2-fold with p<0.05 were classified based on functional category and pathways using GeneSpring GX and Genotypic Biointerpreter-Biological Analysis Software. Pathways that predominantly showed differential expression were (**A**) MAP Kinase pathway, (**B**) Cytokine-cytokine receptor interaction and (**C**) p53 pathway. The data has been submitted to GEO database with accession number GSE50545.

Classification of these genes based on functional category and pathways using GeneSpring GX and Genotypic Biointerpreter-Biological analysis software indicated that the upregulated genes majorly belong to five different pathways (with at least 4 altered genes in each pathway) and the number of genes altered are in the order of Wnt (4 genes)<Focal adhesion (5 genes) <p53 (8 genes)<cytokine-cytokine receptor interaction (11 genes)<MAP kinase pathway (16 genes) (Fig. 3 A–C). Amongst the downregulated genes with at least three genes in each pathway possessing a 2-fold change with a significance of p<0.05 belonged to cytokine-cytokine receptor and MAP kinase pathway (Fig. 3A and B).

At a glance, the obtained results indicate that amongst the altered pathways, MAP kinase signalling pathway appears to be more predominant and significantly affected with nearly 16 altered genes and many of the genes are either upstream or downstream to p38/JNK/ERK pathway such as p53 or regulators of these pathway (Fig. 3A). From the functional point of view, the phosphorylation status of the identified proteins (such as, DUSPs, GADD45A/B, p53 etc) but not merely their transcript levels dictate the cellular fate. Nevertheless, the results obtained indicated the substantial role of MAP kinase signalling and inspired us to focus on the involvement of MAP kinase pathway in embelin induced apoptosis.

### Embelin Induced Activation of p38 and JNK Pathway

**Figure 4:**
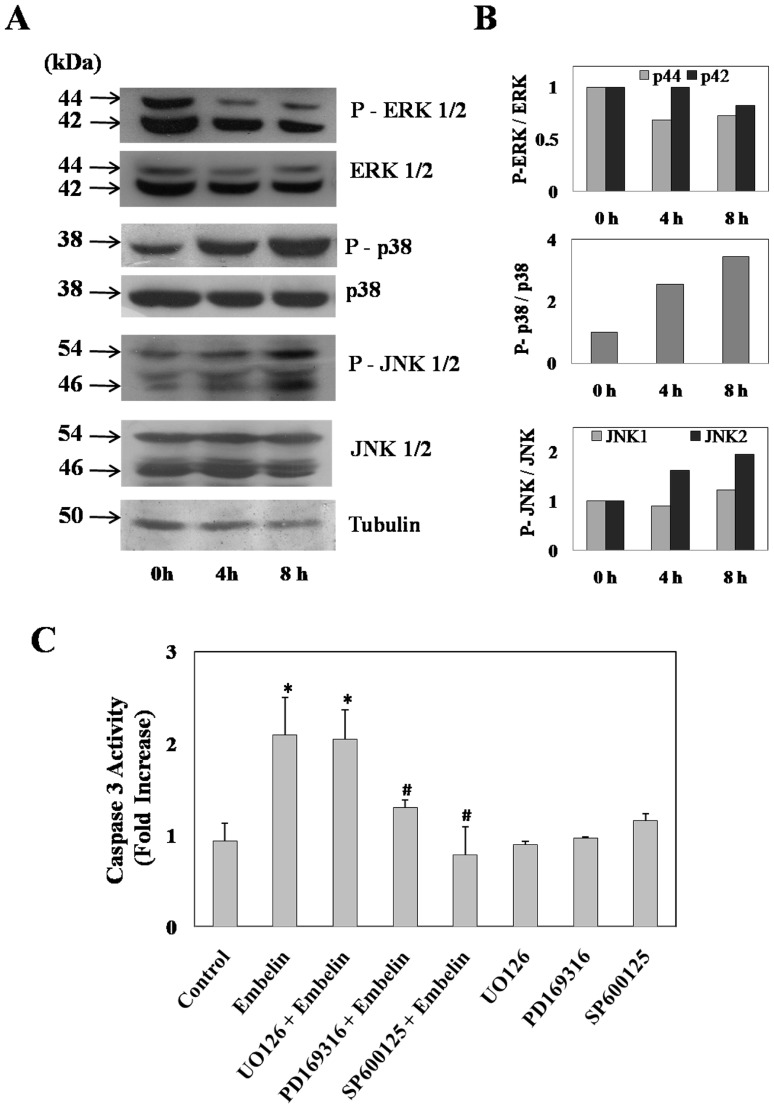
Role of MAP kinases in embelin induced apoptosis. (**A**) A549 cells were treated with 15 µM embelin for 4 and 8h. Total as well as phosphorylated ERK 1/2, p38, JNK 1/2 and tubulin (loading control) levels were measured by Western blot as described in the “Materials and Methods” section. (**B**) Normalized values of the band intensities obtained by densitometric analysis of the data from (**A**). (**C**) Caspase-3 activity in cells pre-treated with or without U0126 (5 µM), PD169316 (5 µM), SP600125 (5 µM) for 1h followed by embelin (15 µM) for 4h. Control values are normalized to 1 and the data indicated are the mean ± SD of three separate experiments. * indicates p<0.05 as compared with control and # indicates p<0.05 as compared with embelin treated cells.

Based on the initial clues obtained from microarray studies on the potential role of MAP kinase pathway in embelin induced apoptosis, we sought to investigate further on the involvement of MAPK signalling and monitored embelin induced alterations in the phosphorylation status of ERK, p38 and JNK proteins (Fig. 4). Results as shown in figure-3 indicate that embelin (15 µM) induced the phosphorylation of p38 to nearly 2.5 and 3-fold by 4 and 8h respectively. Phospho-JNK 1/2 levels were also increased to 1.2 and 1.9 fold respectively by 8h. However, under similar treatment conditions there was a significant decrease in the phosphorylation status of ERK 1/2 (p42 and p44) and the values were found to be 0.3 and 0.2 fold less than that of controls (Fig. 4A and B). In order to understand whether the changes in the phosphorylation status of these MAP kinase proteins has any relevance to the observed apoptosis, we have pretreated cells for 1h individually with specific inhibitors for p38 (PD169316), JNK (SP600125) and MEK (U0126) at 5 µM concentration followed by embelin (15 µM) for 4h. Embelin-induced caspase-3 activity was significantly inhibited by both p38 and JNK inhibitors to nearly control values (Fig. 4C). However, under similar experimental conditions MEK inhibitor (U0126) did not exhibit any protective effect against embelin induced apoptosis and also no significant increase in caspase-3 activity was observed in cells treated with inhibitors alone (Fig. 4C). The observed changes clearly indicate that alterations in the phosphorylation status of both p38 and JNK appears to be crucial in embelin induced apoptosis.

**Figure 5:**
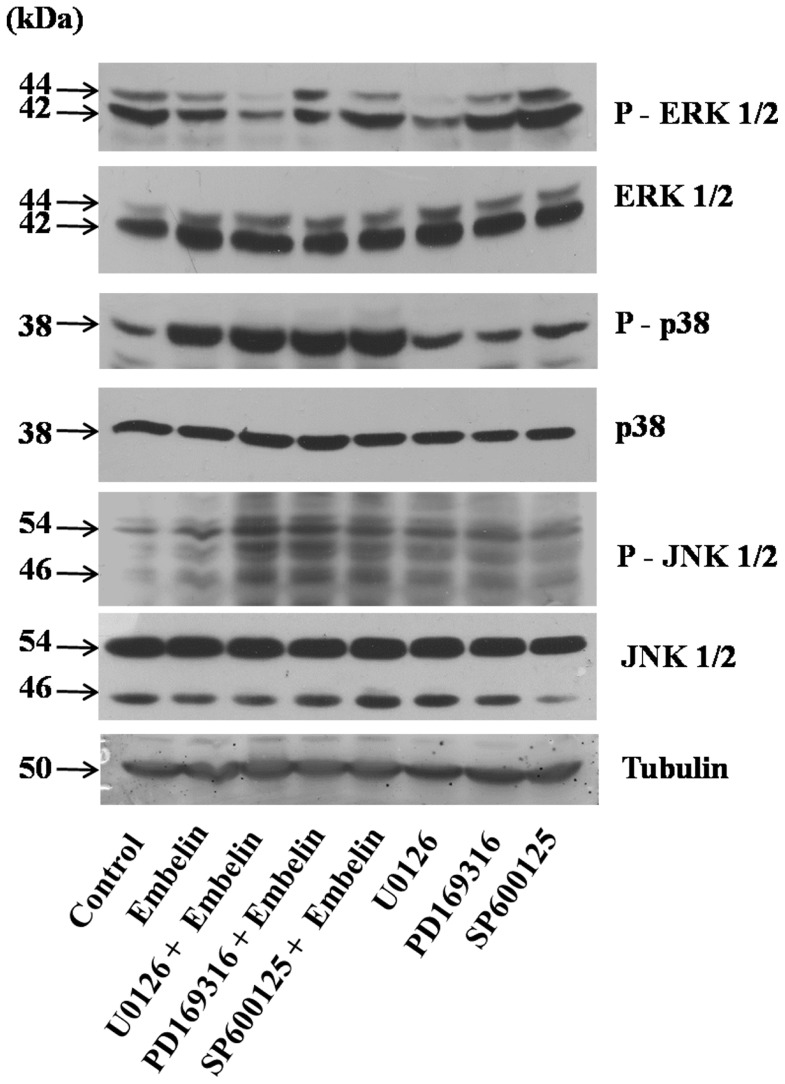
Embelin induced changes in MAP kinase phosphorylation does not involve cross-talk between MAP kinases. A549 cells were pre-treated with or without U0126 (5 µM), PD169316 (5 µM), SP600125 (5 µM) for 1h followed by embelin (15 µM) for 4h. Total and phosphorylated levels of ERK 1/2, p38, JNK 1/2 and tubulin (loading control) were detected by Western blotting as described in the “Materials and Methods” section.

In order to determine whether the observed alterations in the MAPK phosphorylation are because of embelin treatment alone or due to the regulatory effect of one MAP kinase over the other MAPK’s, we have treated the cells individually with embelin (15 µM) in the presence and absence of MEK inhibitor (U0126, 5 µM) or p38 inhibitor (PD169316, 5 µM) or JNK inhibitor (SP600125, 5 µM) for 4h and analyzed the phosphorylation levels of all the three MAP kinases (Fig. 5). The MEK inhibitor U0126 inhibits its downstream target ERK. p38 inhibitor, PD169316 and JNK inhibitor, SP600125 specifically inhibit p38 and JNK activity respectively by competitively binding to the ATP binding pockets preventing the phosphorylation of proteins downstream, but, as such does not result in the decreased phosphorylation levels of either p38 or JNK [26], [27]. Results indicate that treatment of cells with the MEK inhibitor (U0126) inhibited phospho-ERK 1/2, but did not alter the levels of embelin induced phospho-p38 and phospho-JNK levels. Similarly, treatment of cells with p38 inhibitor (PD169316) did not affect the levels of phospho-JNK and phospho-ERK caused by embelin. Also, treatment of cells with JNK inhibitor (SP600125) did not affect the levels of phospho-p38 and phospho-ERK in the presence or absence of embelin (Fig. 5). The above results indicate that the observed changes in the phosphorylation levels of p38, JNK and ERK appears to be directly mediated by embelin treatment, but not due to the cross-talk between the MAP kinases.

### ROS Mediates MAP Kinase Regulation by Embelin

**Figure 6:**
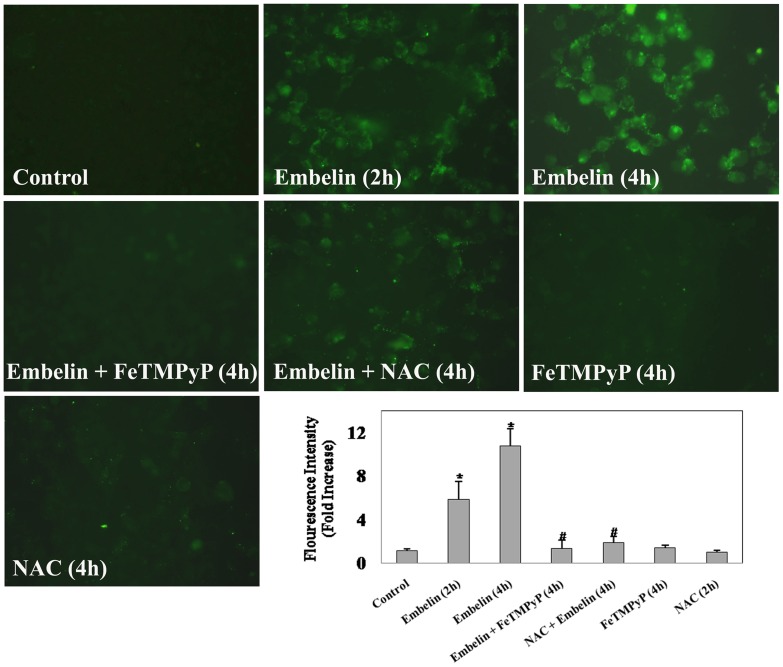
Antioxidants abrogate embelin induced oxidative stress. (**A**) A549 cells were pretreated with or without FeTMPyP (10 µM) or NAC (10 mM) for 1h followed by embelin (15 µM) for 4h and ROS generation was detected by DCF staining as described in the “Materials and Methods” section. Cellular fluorescence was captured using an Olympus–IX71 inverted fluorescence microscope equipped with FITC filter settings. (**B**) Mean fluorescence intensity from three different fields of view were obtained using ImageJ software. * indicates p<0.05 as compared with control and # indicates p<0.05 as compared with embelin treated cells.

MAPK proteins are known to be regulated by oxidative stress [28]. Moreover, the benzoquinone structure of embelin has been demonstrated to form semiquinone radical by redox mechanism which eventually leads to reactive oxygen species generation [13], [29]. These observations suggest an important role for ROS in embelin induced apoptosis. To evaluate the pro-oxidant properties of embelin, we studied its effects on the generation of oxidative stress in A549 cells. The intracellular ROS generated by embelin was detected by an enhancement in the intracellular fluorescence of DCF (Fig. 6). Embelin (15 µM) induced ROS generation in a time dependent manner with nearly 5 and 10-fold increase over untreated controls by the end of 2 and 4h respectively (Fig. 6). Pretreatment of cells with the antioxidant, FeTMPyP (10 µM) or N-acetyl-L-cysteine (NAC) (10 mM) significantly inhibited embelin-induced DCF staining to that of control values (Fig. 6).

**Figure 7:**
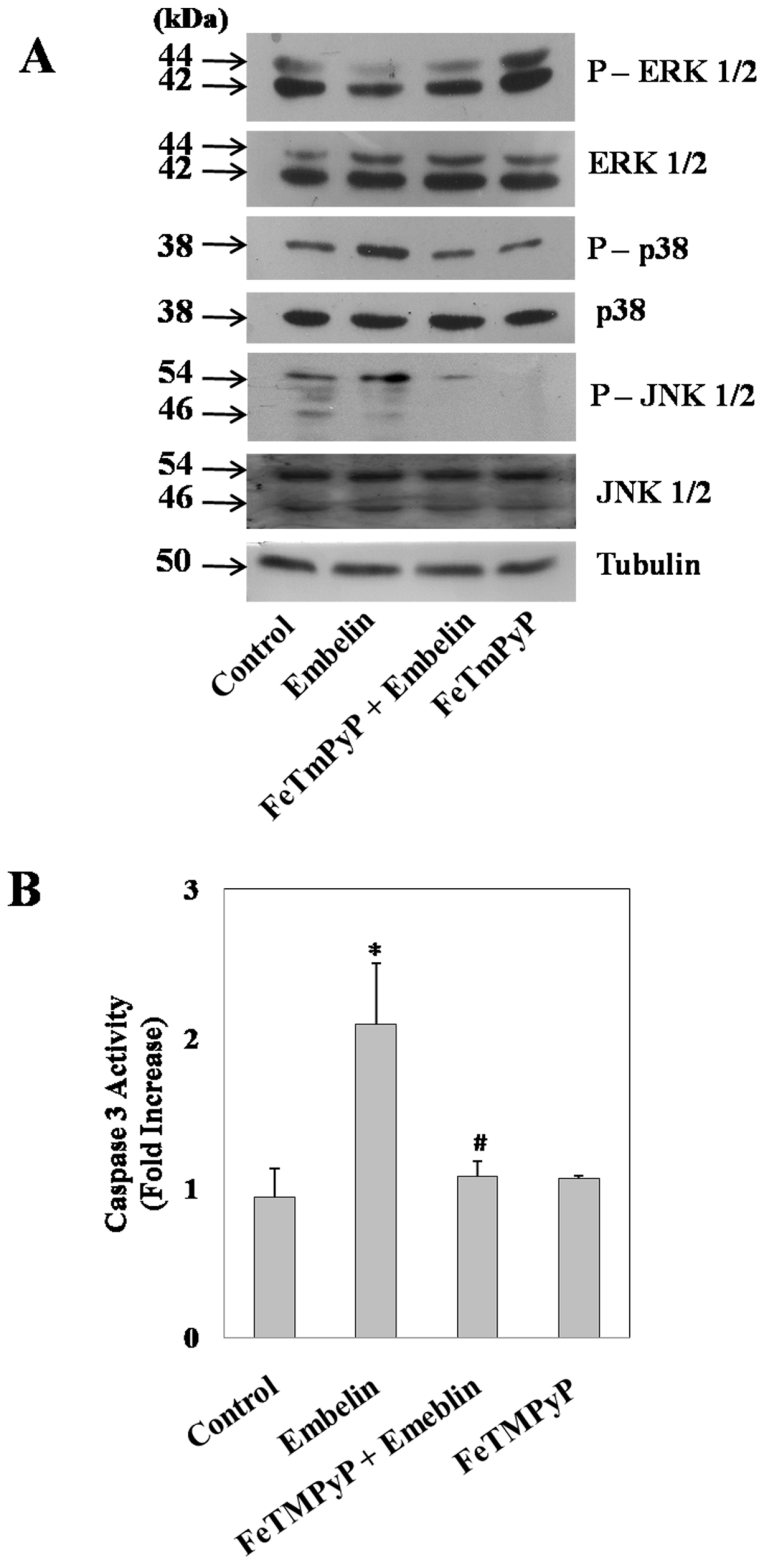
Embelin induced oxidative stress regulates MAPK mediated apoptosis. A549 cells were pre-treated with or without the antioxidant FeTMPyP (10 µM) for 1h followed by embelin (15 µM) treatment for 4h. (**A**) Cellular levels of total and phosphorylated ERK 1/2, p38, JNK 1/2 and tubulin were detected by Western blot followed by chemiluminescence detection as described under “Materials and Methods” section. (**B**) Under similar experimental conditions as (**A**), cellular caspase-3 activity was measured as described under “Materials and Methods” section. Data presented are the mean ± SD of three separate experiments. * indicates p<0.01 as compared to control and **#** indicates p<0.05 as compared to embelin treated cells.

We further assessed the effect of embelin-induced ROS on MAPK signalling in the presence and absence of the antioxidant, FeTMPyP (Fig. 7). Results indicate that embelin induced ROS is responsible for the observed alterations in the phospho-protein levels of p38, JNK and ERK 1/2 as pretreatment of cells with FeTMPyP nullified this effect (Fig. 7A). In accordance with the above results, pretreatment of cells with FeTMPyP (10 µM) also inhibited the apoptotic effects of embelin indicating that altered MAP kinase signalling due to enhanced ROS plays a pivotal role in embelin-induced apoptosis (Fig. 7B).

## Discussion

In the present study, we report that oxidative stress induced MAPK signalling plays an important role in embelin induced apoptosis. Analysis of gene expression profiling by microarray studies indicated the possible involvement of MAP kinase pathway in A549 cells treated with embelin for 4h. Pretreatment of cells with specific inhibitors of either p38 or JNK significantly inhibited embelin induced caspase-3 activation as well as nullified embelin-induced alterations in phosphorylation levels of p38, JNK and ERK 1/2 MAP kinases. Reactive oxygen species (ROS) appears to play a pivotal role between embelin and MAP kinase pathway. All the observed effects of embelin are not due to the inhibition of XIAP as treatment of cells with cell permeable SMAC-N7-Ant peptide, which binds to the BIR3 domain of XIAP did not affect cellular caspase-3 activation.

The natural product, embelin, has been paid more attention in recent times for its anticancer properties. More importantly, it has been demonstrated to have more selectivity towards cancer cells as compared to the normal cells (6). Even in the present work, we observed a similar trend and a significant difference in the IC_50_ values of embelin was evident between lung cancer and normal cell lines (Fig. 1). Though embelin was initially identified to be an inhibitor of XIAP by means of its interaction at BIR3 domain, subsequent studies demonstrated the direct *in vitro* effects of embelin on the oxidative phosphorylation of mitochondria, inhibition of 5-lipoxygenase (5-LO) and microsomal prostaglandin E2 synthase-1 (mPGES)-1 and inactivation of plasminogen activator inhibitor-1 (PAI-1) [10], [11]. However, identification of the primary intracellular target which is responsible for the anticancer property of embelin might eventually help in the structural refinement of embelin for improving its efficacy and selectivity.

Recently, various studies have been carried out to understand the mode of action of embelin and it has been demonstrated to have a role in the inactivation of NF-kB, inhibition of STAT3 signalling via protein tyrosine phosphatase PTEN, lysosomal destabilization and AKT and mTOR pathways [8], [9], [15], [30], [31]. However, whether all the observed effects are interdependent or independent of each other is not yet clear as many of the reported experiments were carried out at a fixed duration of either 24 or 48h [8], [10], [16], [17].

Data from microarray studies during the early stages of embelin induced apoptosis pointed us to the changes in the regulation of transcription factors downstream to MAPK proteins (Fig. 3). In the present study, we have identified a prominent role of MAP kinase pathway, (increased levels of phospho-p38 and phospho-JNK) in embelin-induced apoptosis. All the three MAP kinases are regulated independently by embelin/embelin-induced ROS as none of the specific inhibitors for individual MAP kinases affected the phosphorylation status of other MAP kinases (Fig. 4).

MAPK proteins play a key role in cellular events affecting diverse end points including cell proliferation, differentiation, cell survival and cell death [32]. Phosphorylation of ERK 1/2 decreased in time dependent fashion with embelin treatment (Fig. 4A). ERK 1/2 is activated in response to growth stimuli in cancer and targeting it directly or indirectly is known to cause tumour cell death [32], [33]. In addition, embelin also induced significant elevation in the phosphorylation of p38 and JNK 1/2. JNK, also referred as stress activated protein kinase, is activated by various stress stimuli like changes in osmolarity or metabolism, DNA damage, heat shock, inflammatory cytokines, shear stress, UV irradiation or oxidative stress [32]. p38 in most cases is activated simultaneously with JNK [32]. The anti-apoptotic effects of ERK 1/2 and pro-apoptotic effects of p38/JNK are already described [34]. In accordance with these earlier reports, p38 and JNK inhibitors (PD169316 and SP600125) abrogated embelin-induced apoptosis, while MEK inhibitor (U0126) did not show any significant effect (Fig. 4C). However, these events involving simultaneous down-regulation in the phospho-ERK levels and concomitant activation of p38/JNK pathways during embelin mediated apoptosis are regulated independent of each other (Fig. 5).

Embelin is a benzoquinone with an aliphatic chain which has quinone and hydroquinone groups on the aromatic ring. Because of which, it can either be oxidised or reduced to form a semiquinone radical [13]. Recently, embelin has also been shown to generate intracellular ROS [29]. Even the present study demonstrates an enhancement in cellular ROS generated by embelin as early as 4h and pretreatment of cells with the antioxidants abrogated this effect as well as embelin-induced alterations in phosphorylation status of MAP kinases and apoptosis (Fig. 6 & 7). Very similar to our findings, Allensworth *et al*., reported that SOD mimic MnTnHex-2-PyP^5+^ reversed the toxic effects of embelin when treated in combination with TRAIL in XIAP overexpressed SUM149 cells [29]. Moreover, the lack of cellular caspase-3 activation by SMAC-N7-Ant peptide as shown in figure 2B, clearly indicates that the observed effects of embelin on MAP kinase mediated apoptosis is independent of its interaction at the BIR3 domain of XIAP.

Overall, the present study identified the role of ROS induced alterations in the MAPK signalling pathway, especially activation of p38 and JNK, as responsible mediators in embelin induced apoptosis. The observed effects are not mediated by XIAP inhibition alone as treatment of cells with a known XIAP’s BIR3 domain inhibitor, i.e., SMAC-N7-Ant peptide did not replicate embelin-induced apoptosis. Further studies aimed at enhancing the apoptotic potential or selectivity of embelin towards cancer cell lines in combination with MAP kinase modulators or their downstream targets might lead to novel therapeutic strategies as well as improve the therapeutic efficacy of embelin.
